# fMRI informed voxel-based lesion analysis to identify lesions associated with right-hemispheric activation in aphasia recovery

**DOI:** 10.1016/j.nicl.2022.103169

**Published:** 2022-08-27

**Authors:** Hans R. Schneider, Max Wawrzyniak, Anika Stockert, Julian Klingbeil, Dorothee Saur

**Affiliations:** Neuroimaging Laboratory, Department of Neurology, University of Leipzig Medical Center, Leipzig, Germany

**Keywords:** AAT, Aachen Aphasia Test, ATL, anterior temporal lobe, BNA, Brainnetome Atlas, dlPFC, dorso-lateral prefrontal cortex, DWI, diffusion-weighted imaging, EmC, extreme capsule, EPI, echo-planar imaging, FLAIR, fluid-attenuated inversion recovery, fMRI, functional magnetic resonance imaging, FSL, FMRIB Software Library, FWE, family-wise error, IFG, inferior frontal gyrus, LRScomp, language recovery score for comprehension, MNI, Montreal Neurological Institute, MPRAGE, Magnetization Prepared - Rapid Gradient Echo, MTG, middle temporal gyrus, PTL, posterior temporal lobe, SMA, supplementary motor area, SPM12, Statistical Parametric Mapping, version 12, STG, superior temporal gyrus, TE, echo time, TR, repetition time, ROI, region of interest, VLBM, voxel-based lesion-behavior mapping, Right hemisphere, Aphasia, Language, Voxel-based lesion behavior mapping, VLBM

## Abstract

Several mechanisms have been attributed to post-stroke loss and recovery of language functions. However, the significance and timing of domain-general and homotopic right-hemispheric activation is controversial. We aimed to examine the effect of left-hemispheric lesion location and time post-stroke on right-hemispheric activation. Voxel-based lesion analyses were informed by auditory language-related fMRI activation of 71 patients with left middle cerebral artery stroke examined longitudinally in the acute, subacute and early chronic phase. Language activation was determined in several right-hemispheric regions of interest and served as regressor of interest for voxel-based lesion analyses. We found that an acute to chronic increase of language activation in the right supplementary motor area was associated with lesions to the left extreme capsule as part of the ventral language pathway. Importantly, this activation increase correlated significantly with improvement of out-of-scanner comprehension abilities. We interpret our findings in terms of successful domain-general compensation in patients with critical left frontotemporal disconnection due to damage to the ventral language pathway but relatively spared cortical language areas.

## Highlights

• We examined associations between lesion location and right-hemispheric language activation.
• Lesions to the left extreme capsule were related to longitudinally increasing activity in the right supplementary motor area.
• This activation increase correlated with improvement of out-of-scanner comprehension abilities.
• We interpret our findings in terms of successful domain-general compensation in patients with damage to the ventral language pathway.

## 1 Introduction

Aphasia is one of the most debilitating consequences of stroke (Lazar et al., 2008, Lee et al., 2015, Flowers et al., 2016). In most cases, aphasia substantially improves with time after stroke. Mechanisms supporting recovery from aphasia include: i) recruitment of perilesional tissue (Baker et al., 2010, Szaflarski et al., 2011, Stockert et al., 2020) or compensation by spared tissue belonging to the language network in the left hemisphere (Saur et al., 2006, Fridriksson, 2010, Fridriksson et al., 2012, Stockert et al., 2020), ii) recruitment of contralateral areas in the right hemisphere (Saur et al., 2006, Hope et al., 2017, Lukic et al., 2017, Stockert et al., 2020) and iii) stronger involvement of bilateral domain-general networks (van Oers et al., 2010, Brownsett et al., 2014, Geranmayeh et al., 2014).

In particular, the contribution of the right hemisphere is highly debated with controversial appraisal regarding its maladaptive (Martin et al., 2004, Naeser et al., 2005, Price and Crinion, 2005, Hamilton et al., 2011, Turkeltaub, 2015, Chu et al., 2018) or supportive role in language recovery (Crinion and Price, 2005, Raboyeau et al., 2008, Richter et al., 2008, Schlaug et al., 2011, Robson et al., 2014, Xing et al., 2016). Several studies found that higher magnitude of activation of right hemisphere areas relates to larger left-hemispheric lesion size (Anglade et al., 2014, Skipper-Kallal et al., 2017b). Additionally, it was demonstrated that lesion location is a possible predictor of right-hemispheric activation (Turkeltaub et al., 2011, Skipper-Kallal et al., 2017a). Specifically, lesions of the left inferior frontal gyrus (IFG) were shown to be related to increased homotopic right hemisphere IFG activation. Further meta-analyses, however, do not consistently support these findings (Stefaniak et al., 2021, Wilson and Schneck, 2021). Furthermore, right-hemispheric activation could reflect both, up-regulation of the domain-general network (Brownsett et al., 2014, Davis and Cabeza, 2015) as well as activation in lesion-homologue areas of the language network (Perani et al., 2003, Winhuisen et al., 2005, Turkeltaub et al., 2011, Griffis et al., 2017).

With respect to the time-course, Saur et al. (2006) demonstrated a higher activation of right frontal regions in the subacute phase. In addition to this activation shift to the right hemisphere in the subacute phase, Stockert and colleagues (2020) demonstrated that recruitment of lesion-homologue cortex was only observed in patients with frontal lesions while (bilateral) domain-general networks were recruited in both, patients with frontal and with temporoparietal stroke.

Based on these previous findings, we hypothesize that activation of right-hemispheric regions depends on left hemisphere lesion location and changes over time. To address this hypothesis, we combined voxel based lesion behavior mapping (VLBM; Bates et al., 2003) with an auditory fMRI comprehension paradigm acquired in 71 stroke patients at three different time points post-stroke (acute, subacute and chronic). While VLBM studies relate behavioral deficits to lesion location, there are only few studies analyzing associations between lesion location and individual functional or structural imaging parameters (Warren et al., 2009, Fridriksson et al., 2010, Tyler et al., 2010, Wilson et al., 2016). In the present study, we related fMRI activation in six right hemisphere regions of interest (ROIs) that were previously reported relevant for language recovery to left-hemispheric lesion location.

## 2 Methods

Local ethics committees approved the experimental procedures according to the Declaration of Helsinki. Each participant or a corresponding legal guardian gave written informed consent. Due to the data protection agreement signed by the participants, which was approved by local ethics committees, data cannot be made publicly available. Anonymized data is available upon reasonable request based on a formal data sharing agreement via Dorothee Saur (dorothee.saur@medizin.uni-leipzig.de).

### 2.1 Overview

**Fig. 1:**
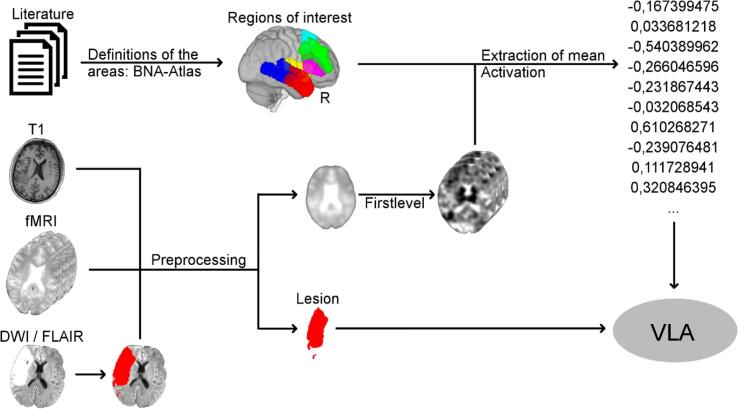
**Overview of the applied methods from raw data to voxel-based lesion analyses.** We performed preprocessing of structural (T1 and DWI/FLAIR) and functional (fMRI) imaging data, lesion delineation and first-level analysis of the fMRI data. Mean right hemispheric language activation from literature-informed ROIs was extracted and related to left-hemispheric lesion locations using voxel-based lesion analyses. These ROIs were defined as IFG (purple), dlPFC (green), SMA (cyan), insular cortex (yellow), ATL (red) and PTL (blue). Used data: structural MRI imaging (T1 for spatial normalization, DWI or FLAIR for lesion delineation), fMRI, BNA atlas maps; numbers represent exemplary extracted mean activation of one distinct BNA atlas based area. DWI, diffusion-weighted image; FLAIR, fluid-attenuated inversion recovery; fMRI, functional magnetic resonance imaging; ROI, region of interest; VLA, voxel-based lesion analysis; BNA atlas, Brainnetome Atlas; R, right.

Fig. 1 displays a brief overview of our methods. We aimed to test for a relationship between lesion location in the left hemisphere and activity (absolute and increase/decrease over time) of several right hemisphere areas of interest. Our analyses were based on lesion masks and longitudinal (acute, subacute and chronic) fMRI language activation data from 71 stroke patients.

We specified six right-hemispheric regions of interest (ROIs) based on prior literature: IFG, anterior temporal lobe (ATL), posterior temporal lobe (PTL), dorso-lateral prefrontal cortex (dlPFC), supplementary motor cortex (SMA) and insular cortex (Turkeltaub et al., 2011, Hartwigsen and Saur, 2019, Stefaniak et al., 2021, Wilson and Schneck, 2021). To analyze the relationship of mean activation in these ROIs and left-hemispheric lesion site, we performed voxel-based lesion analyses (Bates et al., 2003).

### 2.2 Participants and data

We used data from different prior studies from our laboratory (Saur et al., 2006, Saur et al., 2008, Stockert et al., 2020). The same data was also used in a prior study on the resolution of diaschisis (Wawrzyniak et al., 2022). Patients were recruited at three different Departments of Neurology in Germany within a time span of eleven years: the University Medical Center Hamburg-Eppendorf (May 2003–December 2004), the University Medical Center Freiburg (June 2006–March 2010) and the University Medical Center Leipzig (June 2011–May 2014). Inclusion-criteria were left-hemispheric ischemic stroke in the territory of the middle cerebral artery with consequent aphasia, detected by the Aachen Aphasia Test (AAT; Huber et al., 1984). Exclusion criteria were (i) age > 80 years, (ii) too ‘mild’ aphasia (no evidence for aphasia in standard language tests), (iii) preexisting structural brain damage (e.g. infarcts), (iv) native language not German, (v) inability to understand the instructions in the scanner, (vi) recurrent stroke during follow up, (vii) severe small or large vessel disease with hemodynamic infarctions, (viii) claustrophobia, (ix) severe hearing deficits, (x) cerebral hemorrhages, (xi) seizures, (xii) poor imaging quality, (xiii) left-handedness and (xiv) other preexisting neurological or psychiatric diseases. We analyzed data from 71 patients (age 58.0 ± 14.0; 69.0 % male; all right-handed; 13 – Hamburg, 51 – Freiburg, 7 – Leipzig). See SI Table 1 for detailed demographic information. To account for different effect sizes at the different sites, we also selected 14 healthy age- and sex-matched controls (age 57.6 ± 13.6; 57.1 % male) from the different sites (5 – Hamburg, 5 – Freiburg, 4 – Leipzig).

#### 2.2.1 Study design

Patients were examined at three different time points during stroke recovery: in the acute phase 0 to 3 days post stroke (acute, mean 1.8 days), the subacute phase 4 to 22 days post stroke (subacute, mean 10.7 days) and the (early) chronic phase 3 to 10 months after stroke (chronic, mean 150.0 days). At each time point, language performance of the patients was examined using the AAT. Several patients were examined two times or only once. In sum 55 acute, 69 subacute and 53 chronic datasets were available. For 44 patients, complete longitudinal datasets with three time points were available. All control participants were examined only once.

#### 2.2.2 fMRI paradigm

We used fMRI to examine language-specific blood oxygen level dependent signal in the whole brain in an event-related design. To this end, we presented two types of auditory stimuli: Intelligible speech with or without a semantic violation (e.g. ‘The pilot flies the plane./The pilot eats the plane.’ hereafter referred to as ‘Sp’) and unintelligible temporally reversed speech (e.g. ‘.enalp eht stae/seilf tolip ehT’, hereafter referred to as ‘Rev’). Two slightly different fMRI paradigms were used: paradigm 1, consisting of 92 stimuli in each condition (Sp and Rev) equally distributed over 6 sessions and paradigm 2, consisting of 90 stimuli per condition distributed over 3 sessions for the controls while patients completed only a single session with 30 stimuli per condition. Examined patients and controls in Hamburg, Leipzig and 11 in Freiburg underwent paradigm 1, the remaining patients examined in Freiburg underwent paradigm 2.

#### 2.2.3 MRI acquisition

MRI scans were performed on 3 Tesla scanners (Siemens Trio in Hamburg/Freiburg and Siemens Verio in Leipzig). We used an echo planar imaging (*EPI*) sequence (Hamburg: 115 scans per session, repetition time (TR): 1.83 s, echo time (TE): 25 ms, voxel size: 3 mm isotropic; Freiburg: 115 [paradigm II: 260] scans per session, TR: 2.19 s, TE: 30 ms, voxel size: 3 mm isotropic; Leipzig: 115 scans per session, TR: 1.89 s, TE: 25 ms, voxel size: 3 mm isotropic). Additionally, each patient was scanned with a high-resolution magnetization prepared rapid gradient echo T1 imaging (MPRAGE, voxel size 1x1x1 mm³), T2-weighted fluid attenuated inversion recovery (FLAIR) and diffusion weighted imaging (DWI) sequence.

#### 2.2.4 Lesion delineation

Lesions were semi-automatically delineated on the most suited available scan (acute DWI or subacute FLAIR) using the Clusterize Toolbox (Haan et al., 2015). After delineation, lesion masks were manually edited by a neurologist using MRIcron (Rorden and Brett, 2000).

#### 2.2.5 Language testing

All patients were behaviorally characterized using the AAT. This multidimensional test includes formal rating of spontaneous speech (semi-standardized interview), comprehension (token test, auditory picture matching task, reading), repetition (phonemes, monosyllabic words, compound words, loan words, sentences), picture naming (simple objects, colors, compound words, situations) and written language (writing, combining words, reading). Presence of aphasia was determined by a combination of different sub-scores (especially token test).

We computed a language recovery score for comprehension (LRScomp) representing overall comprehension performance at the different time points. The LRScomp was defined as the sum of all AAT comprehension subscores divided by the maximum possible score. This comprehension score was used to match the fMRI comprehension paradigm. Behavioral scores were available from 54/55 acute, 67/69 subacute and 50/53 chronic time points. Missing scores are caused by discontinued testing due to health related reasons. SI Table 2 contains the individual language scores.

### 2.3 Data analysis

#### 2.3.1 Preprocessing

We used Statistical Parametric Mapping (SPM12, v7487, Wellcome Trust Centre for Neuroimaging, London) and in-house tools with Matlab (2018b, MathWorks) for fMRI data preprocessing. We discarded the first four scans, corrected for different slice acquisition times and performed motion correction (two-pass realignment). The lesion source (DWI or FLAIR imaging) and lesion map were coregistered to the individual T1-weighted high-resolution image. Individual lesions of the patients were masked from the high-resolution structural image. Structural imaging (and lesion masks) were then coregistered to the mean functional image and segmented using the unified segmentation approach (Ashburner and Friston, 2005). The resulting nonlinear deformation field was used to spatially normalize and resample the functional scans (3x3x3 mm^3^ voxel size) and lesion masks (1x1x1 mm^3^) to the MNI152 space. Functional imaging data was additionally convolved with an isotropic Gaussian smoothing kernel with full width at half maximum of 8 mm.

#### 2.3.2 First level

First level analysis was performed using a mass-univariate approach based on General Linear Models with SPM. The design matrix included the onsets and durations of the auditory stimuli (Sp and Rev) convolved with the canonical hemodynamic response function and the six realignment parameters (representing in-scanner motion) as covariates of no interest. Additionally, a drift fit with Discrete Cosine Transform basis (128 s cut-off) was applied. Estimated beta images were used to create contrast images, which represent language-specific activation by subtracting unintelligible reversed speech from intelligible speech (Sp > Rev), regardless of semantic violation.

The magnitudes of uncorrected fMRI activation showed a considerable amount of variability between sites/paradigms caused by the different paradigms and MR-scanners at the three research sites. To achieve comparable levels of activations across sites/paradigms, we used a global scaling procedure (Wawrzyniak et al., 2022). A factor proportional to the magnitudes of fMRI activation in the group of healthy controls was calculated for each research site/paradigm separately. This factor was defined as the mean (for every site/paradigm) of the standard deviation (calculated for every control subject separately) across all voxels in the brain for the auditory main effect (Sp + Rev > rest). This factor was additionally adjusted for the number of sessions per patient. All contrast images were then divided by the corresponding factor.

#### 2.3.3 Atlas-based regions of interest

We defined regions of interest in the right hemisphere where language activation in patients with post-stroke aphasia has been reported in the literature. Because we use these ROIs to extract functional activation, we decided to define these using an atlas based on a functional cortical parcellation: the Brainnetome Atlas (Fan et al., 2016). The regions of interest encompass posterior (Xing et al., 2016, Hope et al., 2017) and anterior temporal cortex (Crinion and Price, 2005, Robson et al., 2014; including the middle temporal gyrus (MTG) and the superior temporal gyrus (STG)), inferior frontal gyrus (IFG; Saur et al., 2006, Turkeltaub et al., 2011, Stockert et al., 2020), dorso-lateral prefrontal cortex (dlPFC; Stockert et al., 2020, Stefaniak et al., 2021), insular cortex (Raboyeau et al., 2008, Richter et al., 2008, Brownsett et al., 2014) and supplementary motor cortex (SMA; Saur et al., 2006, Brownsett et al., 2014, Griffis et al., 2017). A complete list of the atlas regions used as ROIs is provided in SI Table 3. The resulting binary masks were used to extract the mean language activation per ROI for each patient for all time points.

#### 2.3.4 Voxel-based lesion analysis

To determine voxel-wise dependencies between lesion location and right hemisphere activation, voxel-based lesion analysis (Bates et al., 2003, Rorden et al., 2007) was performed using a modified version of NiiStat (https://www.nitrc.org/projects/niistat/) for Matlab. We performed separate voxel-based lesion analyses for each ROI and time point. We included right hemisphere activation as regressor of interest and lesion-size as covariate of no interest (Haan and Karnath, 2018) into the General Linear Model. The analysis was restricted to voxels damaged in at least 10 % of all patients (Sperber and Karnath, 2017, Karnath et al., 2018). To control the family wise error rate (FWE), the null-distributions of the maximum z-scores were obtained by 5,000 random permutations of the design matrix using Freedman-Lane procedure and the results were thresholded at p(FWE) < 0.025 (two-tailed approach) on the voxel-level (Nichols and Hayasaka, 2003, Eklund et al., 2016). A two-tailed approach was chosen, because an increase as well as decrease of right hemisphere activation could be associated with the left hemisphere lesion.

In addition to the cross-sectional approach described above, we also analyzed longitudinal changes (i.e. subacute – acute, chronic – subacute and chronic – acute) in language activation of the right-hemispheric ROIs in relation to lesion location in an analogous fashion.

#### 2.3.5 Correlation with behavior

Significant results from the voxel-based lesion analyses indicate an association of left-hemispheric lesion location and time dependent activation in right-hemispheric ROIs. To investigate whether these activations are behaviorally relevant, we tested for linear correlations of activation (or longitudinal activation increase) in the distinct ROI with comprehension performance in terms of LRScomp (or its increase) using Pearson correlation coefficients.

#### 2.3.6 Fiber tracking

Since our main voxel-based lesion analysis result appeared to be localized within the subcortical white matter (see results), we decided to perform DTI fiber tractography using the significant voxel-based lesion analysis results as seed. Fiber tracking was based on diffusion weighted MRI scans (2x2x2 mm³, 128 directions, b-value 1500 s/mm^2^ and additional nine b0-weighted images) and T1-weighted MPRAGE images (1x1x1 mm³, TR/TE: 1900/2.52 ms, flip angle: 9°) of 25 healthy participants (10 females) aged 49 to 64 years from the Enhanced NKI sample (Nooner et al., 2012). Fiber tracking including preprocessing was performed with FSL v6.0 (Jenkinson et al., 2012). Diffusion weighted images were corrected for eddy current-induced distortions and in-scanner motion (Andersson and Sotiropoulos, 2016) and non-brain tissue was deleted (Smith, 2002). We then performed bayesian estimation of diffusion parameters obtained using sampling techniques (Behrens et al., 2007, Jbabdi et al., 2012). A series of linear (DWI to T1-weighted image, T1 to MNI template) and non-linear (T1 to MNI) spatial registrations (Jenkinson and Smith, 2001) and their inversion was performed to be able to transform the region of interest (voxel-based lesion analysis result) into the native DWI space but transform tractography results back to MNI space. Probabilistic tractography as implemented in ‘probtrackx2′ was seeded from the significant voxel-based lesion analysis result with correction of path distributions for the length of the pathways. Path distribution maps were divided by the total number of generated fibers. Finally, we computed a mean of all 25 path distribution maps which was convolved with an isotropic Gaussian smoothing kernel with full-width at half maximum of 3 mm (Saur et al., 2008) and then (arbitrarily) thresholded at > 10 %.

## 3 Results

### 3.1 Lesion distribution

**Fig. 2:**
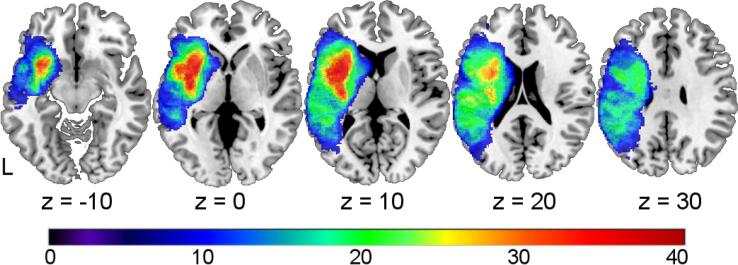
**Lesion overlay (n = 71).** Color indicates number of overlapping lesions (restricted to areas damaged in at least 10% of all patients); number of participants overall: 71; coordinates refer to MNI-space; L, left.

Fig. 2 displays the lesion overlay of all 71 patients which corresponds to the territory of the left middle cerebral artery with highest overlap in subcortical structures.

### 3.2 Voxel-based lesion analysis

We performed fMRI informed voxel-based lesion analyses in 71 stroke patients with aphasia to examine relationships between right-hemispheric language activation and left-hemispheric lesion location. We performed separate voxel-based lesion analyses for six different right-hemispheric ROIs, both in a cross-sectional (acute, subacute, chronic) and longitudinal (subacute – acute, chronic – subacute, chronic – acute) design, resulting in 36 different voxel-based lesion analyses. Table 1 gives an overview of the results from all analyses.

**Table 1:** Associations between left-hemispheric lesion location and right-hemispheric language activation.

Time points/ROIs	rATL	rPTL	rIFG	rdlPFC	rInsula	rSMA
acute (n = 55)	n. s.	n. s.	n. s.	n. s.	n. s.	n. s.
subacute (n = 69)	n. s.	n. s.	n. s.	n. s.	n. s.	n. s.
chronic (n = 53)	n. s.	n. s.	n. s.	n. s.	n. s.	n. s.
subacute – acute (n = 55)	n. s.	n. s.	n. s.	n. s.	n. s.	n. s.
chronic – subacute (n = 53)	n. s.	n. s.	n. s.	n. s.	n. s.	n. s.
chronic – acute (n = 53)	n. s.	n. s.	n. s.	n. s.	n. s.	left subinsular white matter (72 voxels)

This table summarizes all voxel-based lesion analysis results thresholded at p(FWE) < 0.025 (two-tailed tests). Abbreviations: n. s., not significant; rATL, right anterior temporal lobe; rPTL, right posterior temporal lobe; rIFG, right inferior frontal gyrus; rdlPFC, right dorso-lateral prefrontal cortex; rSMA, right supplementary motoric area; left STG, left superior temporal gyrus.

#### 3.2.1 Cross-sectional

There were no significant associations between left-hemispheric lesion location and right-hemispheric fMRI language activation.

#### 3.2.2 Longitudinal

**Fig. 3:**
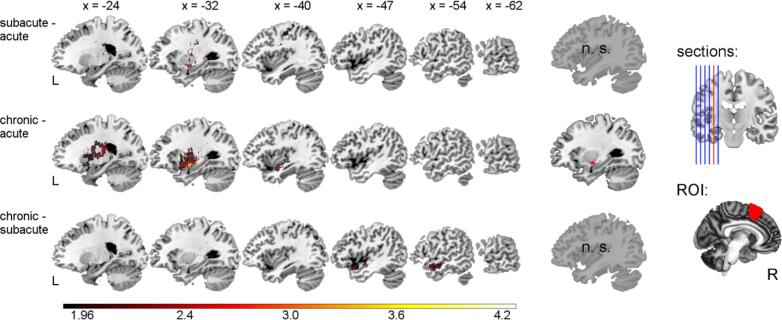
**Association of lesion localization with longitudinal changes of language activation in the right SMA.** Left panel shows longitudinal voxel-based lesion analysis results (subacute > acute, chronic > acute and chronic > subacute; the opposite tails are not displayed here); Z-values are shown at p < 0.05 uncorrected (for display purposes), middle panel shows significant voxels thresholded at p(FWE) < 0.025. Right panel shows location of sagittal sections and location of the ROI in the right SMA; brighter colors (overlays) refer to higher Z-values; x-coordinates refer to MNI-space; ROI = right SMA (right supplementary motor area); n. s., not significant; L, left; R, right.

**Fig. 4:**
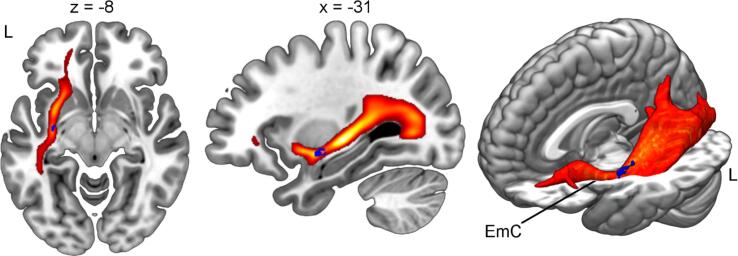
**Fiber tractography seeding from subcortical voxel-based lesion analysis result implicates left extreme capsule.** The subcortical cluster where damage was associated with increasing activity in the right SMA from the acute to the chronic time point (displayed in blue) was used to seed fiber tractography in 25 healthy participants (displayed in warm colors). Color indicates mean relative overlap thresholded at > 10 %. Coordinates refer to MNI space. Abbreviations: EmC, extreme capsule; L, left.

We found a cluster of 72 voxels (peak: MNI = −34/−3/−12 mm, p(FWE) = 0.0012) with an association of lesions in the left subinsular white matter and activation increase from acute to chronic in the right SMA (Fig. 3). Fiber tractography seeding from this subcortical cluster depicted frontotemporal association fibers passing through the left extreme capsule (see Fig. 4).

Supporting our significant result concerning the chronic upregulation of the right SMA, we found a similar subsignificant pattern associating lesions in left subinsular white matter with increased activity of the right SMA in the subacute in contrast to the acute phase (subacute – acute; see Fig. 3). While these findings are reflected in the chronic phase as well (see SI Fig. 1), subtracting subacute from chronic activity showed none of these associations (chronic – subacute; see Fig. 3).

#### 3.2.3 Specificity

We performed random permutation testing to address multiple comparisons across voxels within each analysis. To also account for the fact that we carried out 36 separate analyses, we performed an additional specificity analysis. To this end, we permuted all 36 design matrices simultaneously to obtain the distribution of the maximum test statistic (accounting for the two-tailed nature) across all voxels and analyses at the same time. This enabled us to control the family wise error rate to be < 5 % across all voxels and analyses. We still found 6 voxels in the left extreme capsule where damage was associated with increasing language activity in the right SMA from the acute to the chronic measurement.

**Fig. 5:**
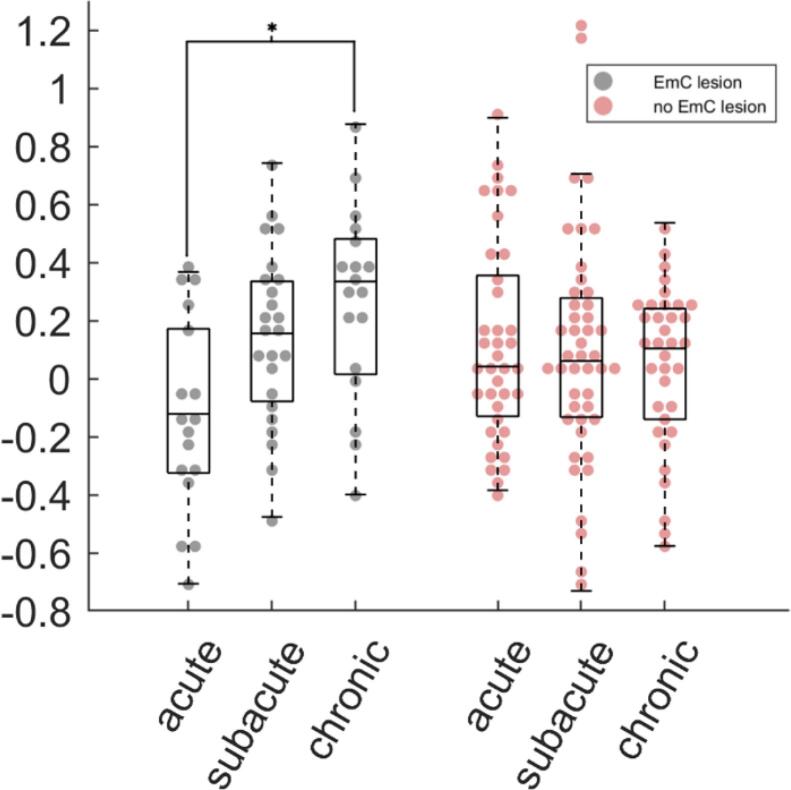
**Longitudinal right SMA language activation.** Mean right SMA fMRI language activation (beta estimates, Sp > Rev, arbitrary units) at the different time points in patients with lesions overlapping (gray dots) or not overlapping (red dots) with the lesion mapping result in the extreme capsule. Central marks of the boxplots represent the median value, the edges are the 25th and 75th percentiles, and the whiskers extend to the most extreme data points not >150% of the interquartile range beyond the boxes. The star indicates a significant difference in activation within group (p < 0.05, Bonferroni corrected).

To further explore the robustness of this result, we plotted right SMA language activity across time separately for patients with and without damage in the extreme capsule (Fig. 5). Damage to the extreme capsule was operationalized by overlap of the individual lesion with the result from the voxel-based lesion analysis (p(FWE) < 0.025, 72 voxels). We additionally analyzed this activation data with a repeated measures ANOVA (n = 44 full longitudinal data sets) with the factors time (acute, subacute, chronic) and group (EmC lesion, no EmC lesion). The between subject factor group did not reach significance (F = 0.15, p = 0.70). However, both the main effect of time (F = 3.80, p = 0.03) as well as the interaction time × group (F = 12.99, p < 0.001) explained a significant amount of variance. We performed post hoc paired t-tests (Bonferroni corrected) between all possible pairs of time points within each group. The only significant finding was an increase from the acute to the chronic time point in the group with EmC lesions (T = 4.51, p < 0.001; see Fig. 5).

#### 3.2.4 Correlation with behavior

**Fig. 6:**
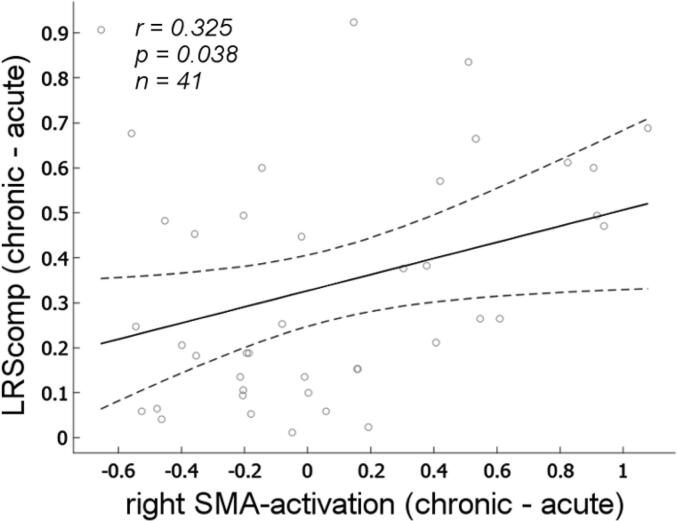
**Correlation of language comprehension performance and language activation.** We examined linear correlations between language comprehension scores and fMRI language activity in the right hemispheric ROI indicated by voxel-based lesion analyses. The graph shows right SMA activation increase (acute to chronic) and the corresponding increase in LRScomp. Circles display individual patients. Solid lines represent best linear fit with its 95 % confidence interval (dashed lines). Abbreviations: r: Pearson correlation coefficient; p: probability value; n: number of patients with available fMRI and behavioral data; right SMA: right supplementary motor area; LRScomp: language recovery score (comprehension).

We found a significant correlation between activation increase in the right SMA from acute to chronic and comprehension score increase from acute to chronic (p = 0.038, r = 0.325; Fig. 6). To explore the specificity of this correlation, we repeated the analysis for the remaining five ROIs. We only found one additional significant correlation between increase in language activation in right IFG and increase in comprehension abilites from acute to subacute (r = 0.41, p = 0.01) but no significent results for the other ROIs.

## 4 Discussion

We performed fMRI informed voxel-based lesion analyses to assess the relationship of left-hemispheric lesion locations and right-hemispheric language activation over the time course of recovery. One out of our 36 analyses revealed a significant result. Lesions that project onto the left extreme capsule were associated with activation increase from the acute to the chronic time point in the right SMA. Additionally, increase in language activation in the right SMA and increase in language comprehension performance from the acute to the chronic phase were significantly correlated. We therefore argue that lesions to the left extreme capsule might be associated with functional recruitment of the right SMA which might support successful language recovery. The extreme capsule is the key fiber tract of the ventral pathway (Petrides and Pandya, 2007). It connects the middle temporal lobe and the ventrolateral prefrontal cortex and subserves language comprehension by mapping sound to meaning (Saur et al., 2008). Damage to this association fiber tract causes frontotemporal disconnection and is associated with comprehension impairments in acute stroke patients (Kümmerer et al., 2013). Our data shows that disruption of this crucial part of the ventral stream evokes up-regulation of right SMA. Increased activity in this region during language recovery has been reported in several previous studies in the subacute (Karbe et al., 1998, Saur et al., 2006) and chronic phase (Brownsett et al., 2014, Griffis et al., 2017). The SMA (particularly the anterior portion) is part of the domain-general network (Hertrich et al., 2016, Cona and Semenza, 2017) and might contribute to language recovery by means of compensatory reallocation of cognitive resources (Brownsett et al., 2014, Geranmayeh et al., 2014). In addition to the relationship between damage to the extreme capsule and increasing SMA activity, we could also demonstrate an association of increasing SMA activity and behavioral improvement. This relationship seemed to be specific because, besides right IFG, it was only found for the right SMA but not for the other ROIs. We therefore argue that lesions to the left extreme capsule might lead to critical disconnection between frontal and temporal (domain-specific) language areas causing comprehension deficits. As these domain-specific cortical hubs might be structurally intact (illustrated by SI Fig. 2), domain-specific compensation mechanisms (e.g. perilesional or contralesional recruitment) might be less efficient due to missing redundancy for the ventral pathway. Instead, impaired integration between these disconnected language-specific hubs might be successfully compensated by stronger activation of domain-general networks which are connected to the domain-specific regions via different fiber pathways. This argumentation is in line with previous findings of bilateral SMA activity increase from the acute to the subacute phase which was correlated with behavioral recovery (Saur et al., 2006). The SMA might support sequential integration of language representations in these patients (Cona and Semenza, 2017). The novel contribution of our study is to spatially localize the critical fiber tract whose damage might trigger this domain-general compensatory mechanism.

Since SMA is also thought to be involved in motor control (Cieslik et al., 2015) and motor learning (Hardwick et al., 2013), some of the activity observed in our fMRI data might be associated with the motor task (button press) in the scanner. However, it is unlikely that the dynamic of SMA activation in the course of language recovery is triggered by the motor task in the scanner paradigm, because increases in motor related activation would not be expected to correlate with language recovery measured outside the scanner using thorough behavioral testing with the AAT.

### 4.1 Limitations

Aim of our study was to identify lesion locations in the left hemisphere that are associated with time-dependent right-hemispheric language activation. Based on previous findings (Stockert et al., 2020), we expected lesion-homologue activation in particular after left frontal lesions. However, besides the highly plausible relationship between activation increase in the right SMA and lesions to the left EmC, no other associations could be identified. In the following we would like to discuss potential limitations and methodological issues of our study which might be responsible for these missing effects.

It is recommended in voxel-based lesion analysis to always correct for effects of lesion size (Haan and Karnath, 2018, Karnath et al., 2018). We therefore included lesion size as a regressor of no interest in our voxel-based lesion analyses as well. Our results therefore implicate regions which are associated with right hemispheric activation beyond the effects of lesion size. However, also repeating our analyses without correction for lesion volume did not uncover additional activation-lesion relationships.

Although we think that our approach of using task-based fMRI activation as regressor of interest has high potential for interpretation of fMRI activation in the lesioned brain, it also harbors some intrinsic problems. In contrast to commonly used behavioral datasets, which offer the possibility of maximizing statistical power by the use of appropriate and validated behavioral tests, a lower signal-to-noise ratio must be assumed for fMRI data. Imaging data comes with physiological noise due to breathing, pulsating vessels and motion artifacts, inter-individual anatomical variability and asynchronously scanned slices. In the process of preprocessing, some of these effects are eliminated (e.g. by slice-time or motion-correction). However, relevant amounts of temporal and spatial noise remain. We therefore speculate that larger group sizes are necessary when combining fMRI and voxel-based lesion analyses compared to common voxel-based lesion behavior mapping.

Another issue that may have introduced noise to our fMRI activation is the inclusion of patients from three different study sites with different scanners and even two slightly different fMRI paradigms. We aimed to correct for these differences by applying a global scaling procedure. Due to small sample sizes of the different sites, it is, however, arguable that we might have partially corrected not only for differences in study site and paradigms but also for real effects.

A further explanation for missing effects (which is not specific to our study) might be the ‘partial injury problem’ (Rorden et al., 2009). It is not clear if the examined behaviors are only caused by one distinct lesion location or different locations. This implies that patients with lesions in different regions could show the same patterns of altered right-hemispheric activation leading to smaller effect sizes. Another general problem of voxel-based lesion analyses is the anatomical architecture of the vessel tree. Regions in the core of the vascular territory of the middle cerebral artery are more often affected by stroke than regions in the periphery (c.f. Fig. 2). This results in poorer statistical power in peripheral regions. In our study, this might explain the failure to demonstrate an association of right-hemispheric activation with left cortical lesions due to less lesion overlap in temporal and frontal core language areas compared to the high subcortical lesion overlap. A related issue is the spatial dependency of examined voxels. Following the artery tree, a proximal occlusion affects proximal and distal voxels, which annihilates the independence of every voxel from each other and leads to spatial displacement towards the center of the vascular territory (Mah et al., 2014, Sperber and Karnath, 2018). These limitations might be partially addressable by multivariate voxel-based lesion analyses in future studies (Karnath et al., 2018).

### 4.2 Conclusion

Combining voxel-based lesion analyses and a longitudinal fMRI language paradigm, we were able to gain insights into dependencies of language activation dynamics in the right hemisphere and lesion location in the left hemisphere. We found that damage to the extreme capsule – which is the bottleneck of the ventral language processing stream – is associated with an upregulation of the right SMA over the course of recovery which might support successful language recovery. We interpret this finding in terms of successful domain-general compensation in patients with critical ventral stream disconnection but relatively spared cortical language areas. Considering the pitfalls related to voxel-based lesion behavior mapping and the potentially low signal-to-noise ratio of fMRI-data in contrast to commonly used validated behavioral tests, larger groups sizes and use of multivariate voxel-based lesion analyses might be useful in future studies combining fMRI and voxel-based lesion analyses.

## Funding

Dorothee Saur and Julian Klingbeil (SA 1723/5-1) are supported by the 10.13039/501100000008Deutsche Forschungsgemeinschaft. Max Wawrzyniak is supported by the Clinician Scientist Program of the Medical Faculty of the University of Leipzig. Open access publication is supported by the University of Leipzig. Hans R. Schneider was supported by a scholarship (‘Promotionsförderung’) of the Medical Faculty of the University of Leipzig.

## CRediT authorship contribution statement

**Hans R. Schneider:** Software, Formal analysis, Writing – original draft, Writing – review & editing. **Max Wawrzyniak:** Investigation, Methodology, Software, Formal analysis, Writing – original draft, Writing – review & editing. **Anika Stockert:** Investigation, Writing – review & editing. **Julian Klingbeil:** Investigation, Writing – review & editing. **Dorothee Saur:** Conceptualization, Investigation, Writing – review & editing.

## Declaration of Competing Interest

The authors declare that they have no known competing financial interests or personal relationships that could have appeared to influence the work reported in this paper.

## Data Availability

Anonymized data is available upon reasonable request based on a formal data sharing agreement via Dorothee Saur (dorothee.saur@medizin.uni-leipzig.de).
