# Supplementary material for: fMRI informed voxel-based lesion analysis to identify lesions associated with right-hemispheric activation in aphasia recovery
Source: Neuroimage Clin. 2022 Aug 27;36:103169. doi: 10.1016/j.nicl.2022.103169 (PMC9440420; doi:10.1016/j.nicl.2022.103169)
Supplement: Supplementary data 1 [file mmc1.pdf]

Supplementary Information

Cross sectional analysis

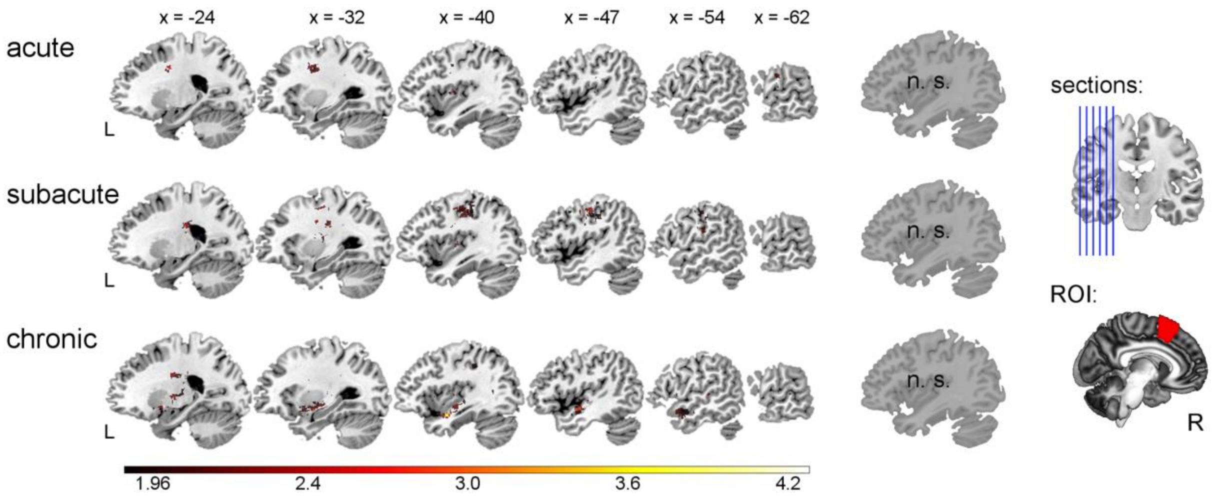

SI Figure 1. Association of lesion localization with language activation in the right SMA at different time points. Left panel shows VLBM results of the acute, subacute and chronic time points; Z-values are shown at $p < 0.05$ uncorrected (for display purposes). Middle panel shows significant voxels thresholded at p(FWE) < 0.025. Right panel shows location of sagittal slices and location of right SMA as region of interest (ROI); brighter colors refer to higher Z-values; x-coordinates refer to MNI-space; n. s., not significant; ROI = right SMA (right supplementary motor area); L, left; R, right.

Subtraction analysis

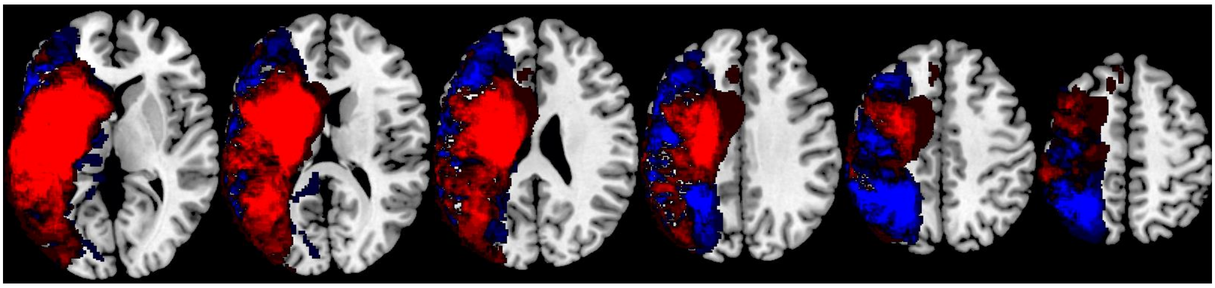

SI Figure 2. Subtraction analysis between patients with and without damage to the extreme capsule. Relative lesion overlap maps of patients with (EmC, n = 25) or without (NonEmC, n = 46) a lesion in the extreme capsule were subtracted from each other. Damage to the extreme capsule was operationalized by overlap of the individual lesion an the voxel-based lesion analysis result. Regions with a higher relative lesion load in the EmC group are displayed blue (mostly cortical regions), with a higher relative lesion load in the NonEmC group are displayed red (preferentially subcortical regions). Darker colors indicate values closer to 0. MNI-coordinates in mm from left to right: z = 5, 15, 25, 35, 45, 55.

Demographic information

SI Table 1. Demographic information. Patients labeled HH, FR_HH and LE were examined with paradigm I, patients labeled FR_FR were examined with paradigm II. Numbers denoted in the columns acute, subacute and chronic refer to days post stroke. Age is given in years at stroke onset. Abbreviations: m = male, f = female, FR_* = Freiburg, HH = Hamburg, LE = Leipzig.

ID	site	age	sex	lesion	lesion volume (cc)	acute	subacute	chronic
1	FR_FR	69	m	frontoparietal	70.4	2	8	129
2	FR_FR	71	m	temporofrontal, subcortical	92.9	2	14	203
3	FR_FR	66	m	parietal	18.7	2		
4	FR_FR	66	m	parietal	34.8	2	5	
5	FR_FR	63	m	parietotemporal	57.6	2	12	109
6	FR_FR	61	f	frontal, temporal, subcortical	71.8	1	13	173
7	FR_FR	76	m	frontal	17.8		8	
8	FR_FR	80	m	occipital	5.2		22	
9	FR_FR	69	m	parietal	19.2	2	10	103
10	FR_FR	34	m	parietotemporal	18.4	1	13	93
11	FR_FR	55	m	parietal	9.3	2	9	89
12	FR_FR	72	f	frontal	87.4		12	132
13	FR_FR	40	f	frontoinsular	40.9	2	10	163
14	FR_FR	78	f	parietal	93.7	3	10	
15	FR_FR	66	f	frontal, subcortical	159.6	3	13	100
16	FR_FR	64	m	frontoinsular	13.6	2	8	105
17	FR_FR	58	m	temporoparietal	4.4	1	8	122
18	FR_FR	71	m	frontotemporoparietal, subcortical	83.7		7	104
19	FR_FR	62	f	frontoinsular, subcortical	43.6		11	332
20	FR_FR	59	m	parietotemporal	23.9	1	8	121
21	FR_FR	31	m	temporoparietal	7.2	1	8	122
22	FR_FR	65	m	frontotemporoparietal	270.9		12	
23	FR_FR	80	f	frontoinsular	24.0		6	
24	FR_FR	61	m	frontal	52.5	2	10	104
25	FR_FR	69	f	frontal	13.6	3	16	
26	FR_FR	49	f	frontal, temporal	43.1	3		
27	FR_FR	68	f	frontal	41.0	1	9	119
28	FR_FR	57	m	parietal, frontal	46.0		12	696
29	FR_FR	80	m	temporal, subcortical	4.4	2	12	
30	FR_FR	58	m	thalamus	4.3	3	12	
31	FR_FR	51	m	frontoinsular	46.3	1	12	110
32	FR_FR	38	m	parietotemporal	9.4	2	10	128
33	FR_FR	69	m	frontal	110.9	2	12	90
34	FR_FR	55	f	frontal, subcortical	67.0		11	
35	FR_FR	72	m	parietotemporal	5.9	1	14	
36	FR_FR	56	m	frontoparietal	184.4	3	8	99
37	FR_FR	63	m	temporoparietofrontal	113.1	3	12	
38	FR_FR	49	m	temporoparietofrontal	136.4	3	11	141

39	FR_FR	68	f	parietotemporal	69.8	2	11	245
40	FR_FR	41	m	frontal, temporal	45.8	2	6	292
41	FR_HH	76	m	parietotemporal	88.7	2	12	100
42	FR_HH	55	m	temporoparietal	132.9	2	8	95
43	FR_HH	69	m	frontal, subcortical	62.6		9	
44	FR_HH	64	m	temporal	15.6	2	8	270
45	FR_HH	73	f	frontal	134.4		14	100
46	FR_HH	61	m	temporoparietal	15.9	1	10	91
47	FR_HH	38	f	frontal, temporal, subcortical	68.1	2	4	236
48	FR_HH	44	m	temporoparietal	20.5	3	10	
49	FR_HH	73	f	temporal	40.5		12	
50	FR_HH	44	f	frontoinsular	19.9	1	9	219
51	FR_HH	68	m	parietotemporal	22.6	0	10	92
52	HH	65	m	frontal	34.4	2	15	220
53	HH	58	f	subcortical	23.1	1	15	182
54	HH	55	m	frontal, parietal, temporal	101.4	2	12	174
55	HH	66	m	frontoparietal	54.0	3	13	
56	HH	65	m	subcortical, parietal, temporal	20.4	1	13	174
57	HH	55	m	temporal	33.1	1	8	92
58	HH	37	m	temporoparietal	33.8	2	10	96
59	HH	57	m	temporal	4.3	1	10	188
60	HH	16	m	frontal, subcortical	64.0	1	11	84
61	HH	43	m	parietotemporal	71.6	1	9	111
62	HH	39	f	temporoparietal, subcortical	155.5		9	253
63	HH	64	f	subcortical, parietal	34.9	2	13	137
64	HH	68	m	frontoparietal	58.2	0	10	104
65	LE	26	f	frontoparietal	99.6		11	89
66	LE	29	m	parietotemporal, frontal	14.6	3	10	122
67	LE	52	m	frontoinsular, subcortical	26.5		12	98
68	LE	40	f	parietal	19.0	2	10	101
69	LE	50	f	frontal	67.9		13	111
70	LE	49	m	temporoparietal	26.3	2	13	91
71	LE	61	m	temporal	11.3	1	10	96

Behavioral data

SI Table 2. Behavioral data. LRScomp = Language recovery score comprehension (0-1). LRSprod = Language recovery score production. n.a. = not available.

ID	acute		subacute		chronic	
	**LRScomp**	**LRSprod**	**LRScomp**	**LRSprod**	**LRScomp**	**LRSprod**
1	0.51	0.00	0.58	0.09	0.96	0.83
2	0.17	0.27	0.55	0.72	0.85	0.88
3	0.71	0.66				
4	0.23	0.44	0.58	0.61		
5	0.94	0.11	0.97	0.70	0.98	0.87
6	0.02	0.00	0.41	0.44	0.69	0.67
7			0.61	0.67		
8			0.18	0.60		
9	0.84	0.90	0.94	0.98	0.97	0.94
10	0.98	0.91	0.98	0.98	0.99	0.99
11	0.83	0.68	0.92	0.82	0.96	0.92
12			0.41	0.50	0.75	0.81
13	0.27	0.06	0.44	0.55	0.87	0.89
14	0.25	0.35	0.34	0.67		
15	0.03	0.00	0.67	0.73	0.95	0.88
16	0.98	0.90	1.00	0.94	1.00	0.96
17	0.94	0.92	1.00	0.95	1.00	0.97
18			0.34	0.08	0.63	0.44
19			0.69	0.68	0.91	0.97
20	0.73	0.83	0.89	0.93	0.92	0.93
21	0.83	0.88	0.92	0.94	0.98	0.98
22			0.10	0.00		
23			0.06	0.00		
24	0.02	0.00	0.42	0.75	0.93	0.89
25	0.74	0.68	0.85	0.78		
26	0.30	0.27				
27	0.34	0.61	0.39	0.71	0.55	0.81
28			0.62	0.93	0.88	0.97
29	0.36	0.52	0.51	0.56		
30	0.32	0.52	0.79	0.67		
31	0.43	0.51	0.68	0.78	0.88	0.94
32	0.92	0.94	0.98	0.98	0.98	0.98
33	0.34	0.10	0.66	0.77	0.94	0.93
34			0.74	0.93		
35	0.42	0.61	0.58	0.80		
36	0.01	0.00	0.01	0.00	0.49	0.00
37	0.02	0.00	0.10	0.00		
38	0.42	0.00	0.55	0.01	n.a.	n.a.
39	n.a.	n.a.	n.a.	n.a.	n.a.	n.a.
40	0.51	0.29	n.a.	n.a.	n.a.	n.a.
41	0.37	0.68	0.56	0.84	0.75	0.87
42	0.39	0.32	0.46	0.48	0.66	0.62
43			0.33	0.39		
44	0.91	0.86	0.97	0.93	0.98	0.93

45			0.04	0.28	0.49	0.70
46	0.13	0.16	0.59	0.76	0.82	0.87
47	0.84	0.91	0.95	0.97	0.99	0.99
48	0.00	0.11	0.15	0.15		
49			0.72	0.76		
50	0.80	0.83	0.97	0.99	0.98	0.94
51	0.60	0.00	0.79	0.71	0.85	0.84
52	0.40	0.73	0.45	0.81	0.89	0.94
53	0.45	0.71	0.76	0.90	0.92	0.98
54	0.15	0.49	0.40	0.78	0.76	0.96
55	0.34	0.46	0.51	0.54		
56	0.46	0.44	0.91	0.86	0.96	0.94
57	0.52	0.63	0.65	0.72	0.90	0.89
58	0.76	0.51	0.92	0.90	0.96	0.94
59	0.86	0.87	0.93	0.93	0.96	0.94
60	0.42	0.74	0.87	0.95	0.99	1.00
61	0.00	0.00	0.40	0.02	0.84	0.65
62			0.36	0.02	0.52	0.54
63	0.30	0.69	0.34	0.72	0.56	0.92
64	0.46	0.66	0.60	0.90	0.65	0.96
65			0.65	0.89	0.98	0.99
66	0.91	0.91	0.93	0.93	0.96	0.94
67			0.86	0.96	0.95	0.99
68	0.81	0.88	0.84	0.99	0.91	0.98
69			0.47	0.09	0.72	0.76
70	0.58	0.57	0.62	0.70	0.83	0.93
71	0.60	0.79	0.71	0.92	0.71	0.86

ROI definitions

SI Table 3. Right-hemispheric regions of interest and atlas label IDs. ROIs labeled as in the manuscript with the corresponding label IDs by Fan et al. 2016. Abbreviations: IFG = inferior frontal gyrus, dlPFC = dorsolateral prefrontal cortex, SMA = supplemental motor area, PTL = posterior temporal lobe, ATL = anterior temporal lobe, ROI = region of interest.

**ROI name**	**Label IDs**	**note**
IFG	30 (IFG_R_6_1), 32 (IFG_R_6_2), 34 (IFG_R_6_3), 36 (IFG_R_6_4), 40 (IFG_R_6_6)	
Insula	164 (INS_R_6_1), 166 (INS_R_6_2), 168 (INS_R_6_3), 170 (INS_R_6_4), 172 (INS_R_6_5), 174 (INS_R_6_6)	
dlPFC	16 (MFG_R_7_1), 18 (MFG_R_7_2), 20 (MFG_R_7_3), 22 (MFG_R_7_4), 24 (MFG_R_7_5)	
SMA	2 (SFG_R_7_1)	
PTL	72 (STG_R_6_2), 74 (STG_R_6_3), 76 (STG_R_6_4), 80 (STG_R_6_6), 82 (MTG_R_4_1), 86 (MTG_R_4_39), 88 (MTG_R_4_4)	Divided manually into anterior and posterior portion
ATL	70 (STG_R_6_1), 74 (STG_R_6_3), 78 (STG_R_6_5), 80 (STG_R_6_6), 82 (MTG_R_4_1), 84 (MTG_R_4_2), 88 (MTG_R_4_4)	
